# Quantifying Subject-Identity Variance in Spectral EEG Features and Its Role in Machine Learning Evaluation Leakage

**DOI:** 10.3390/s26196180

**Published:** 2026-09-29

**Authors:** Hassan Ugail, Richard Wirt, Newton Howard

**Affiliations:** 1 Centre for Visual Computing and Intelligent Systems, University of Bradford, Bradford BD7 1DP, UK; 2 ni2o, 3614 Prospect St NW, Washington, DC 20007, USA; richard.wirt@ni2o.com; 3 School of Individualized Study, Rochester Institute of Technology, Rochester, NY 14623, USA; nhmit@me.com

**Keywords:** EEG biometrics, subject identity, latent factor, evaluation leakage, longitudinal EEG, intraclass correlation, variance decomposition, M/EEG methodology, major depressive disorder, wearable EEG

## Abstract

Electroencephalography (EEG) is widely used to study cognitive states and clinical biomarkers, yet the contribution of stable between-subject differences to common EEG feature representations is rarely quantified directly or incorporated into evaluation design. Here, we examine subject-linked structure in spectral EEG features using a longitudinal four-session dataset spanning 199 days and replicate key findings across four public datasets covering 39 to 395 participants, multiple paradigms, and recording systems ranging from low-channel consumer devices to research-grade EEG. In spectral band-power representations, subject identity accounted for substantially more variance than session-related drift and supported extremely strong individual discriminability under subject-wise held-out protocols, with high verification performance and robust cross-session recognition over months. However, this identity structure was not fully invariant across paradigms, showing stronger transfer in richer, higher-channel recordings than in low-channel consumer EEG under larger task shifts. We further show that the same subject-linked structure can inflate downstream clinical classification when evaluation is performed with naive segment-level splits, demonstrating that apparent diagnostic performance can partly reflect identity leakage rather than biomarker learning. These findings highlight subject identity as a measurable and persistent source of structured variance in spectral EEG features and support routine use of subject-wise evaluation and explicit confound diagnostics in EEG machine-learning studies.

## 1. Introduction

Electroencephalography (EEG) and magnetoencephalography (MEG) are widely used to study brain function in cognitive, clinical, and translational settings [1,2]. As machine learning methods have become more common in M/EEG analysis, so too has the need for evaluation procedures that distinguish genuine task- or disease-related signal from other structured variation present in the data. Recent methodological guidance has emphasised that reproducible M/EEG research depends not only on model choice and predictive accuracy, but also on careful treatment of confounds, transparent split logic, and clear reporting of sources of variance that may shape model performance [3,4]. Among these sources, subject-level effects are especially important because M/EEG recordings often contain stable between-person differences that persist across repeated measurements and can be exploited by predictive models, even when identity is not the intended target of analysis.

This issue is easy to overlook because subject specificity in EEG has most often been studied in a different literature, namely, EEG biometrics and brain fingerprinting. That work has shown that spectral features, event-related responses, and connectivity measures can identify individuals with high accuracy across sessions and recording conditions [5,6,7,8,9,10]. Related findings in functional MRI have similarly shown that subject-specific brain signatures can be strong enough to support individual identification [11]. These studies make clear that neurophysiological recordings carry stable person-linked information. However, the methodological implications of that fact for routine EEG machine learning remain underdeveloped. In particular, the strength of subject-linked variance relative to other sources of variation in common EEG feature spaces has rarely been quantified directly, and its relationship to evaluation leakage in downstream decoding or clinical classification has not been established in a unified way.

This gap matters because many EEG machine learning studies are framed as predicting cognitive state, disease status, or behaviour, while the feature representation may also encode who the subject is. When that subject-linked structure is allowed to cross the train/test boundary, a model may achieve high apparent performance by learning stable person-specific signatures rather than the scientific signal of interest. The resulting inflation is not simply a technical nuisance. It affects how results should be interpreted, how strongly claims can be made about brain-based biomarkers or decoding, and whether apparent generalisation is methodologically valid. Although concerns about subject leakage have been raised in several EEG application areas [12,13,14], the field still lacks a clear quantitative account of how large the subject-linked component can be a widely used EEG feature representations, how persistent it is over time and across conditions, and how directly it can distort downstream evaluation.

The present study addresses that problem by treating subject identity as an explicit object of measurement in spectral EEG feature space. Rather than asking only whether EEG can identify individuals, we ask how much of the structured variance in common spectral features is subject-linked, how stable that structure is across sessions and paradigms, and what it implies for evaluation in EEG machine learning. This framing is intended to connect two lines of work that are usually discussed separately. The biometric separability on the one hand, and M/EEG evaluation validity on the other. The core premise is that these are not independent issues. The same stable individual structure that enables EEG fingerprinting can also act as a confound when the scientific task is not identification.

To address this, we analyse four public EEG datasets spanning 39 to 395 participants, multiple paradigms, and acquisition systems ranging from low-channel consumer devices to research-grade recordings. We first quantify subject-linked variance in a longitudinal spectral feature representation using a two-way variance decomposition, and compare it with session-related drift. We then test whether the same structure supports biometric separability under strict subject-wise held-out protocols, how well it persists across sessions over months, and whether it transfers across paradigm changes. Finally, we examine the practical consequence for downstream inference by comparing naive segment-level and correct subject-wise evaluation in a clinical classification setting. Across these analyses, our aim is not to argue that all EEG variability is dominated by identity in every representation, but to show that in common spectral feature spaces subject-linked variance is large enough, stable enough, and predictive enough to require explicit consideration in M/EEG study design and interpretation.

Our contribution is therefore methodological. We show that, in the spectral band-power representations studied here, subject identity is a measurable and often dominant source of structured variance. That this structure is sufficiently persistent to support strong within- and cross-session individual discriminability. That it can materially inflate downstream machine-learning performance when evaluation is not subject-wise. These findings position subject-linked variance not as a background detail, but as a central part of the risk structure of EEG machine-learning studies using comparable features. More broadly, they suggest that evaluating an EEG model requires not only asking whether it predicts well, but also asking what stable between-subject structure in the representation may have enabled that prediction.

### 1.1. EEG Individual Fingerprinting and Biometric Separability

The observation that EEG contains stable person-specific information is well established and has been reviewed across spectral, temporal, and task-evoked approaches spanning multiple acquisition settings [15]. Early brainprint studies showed that event-related potentials can serve as identifying signatures [5], and later work extended this idea to spectral and connectivity-based representations across resting-state, naturalistic, and task settings. Demuru and Fraschini [6] showed that the aperiodic spectral component provides robust subject discrimination, while Fraschini and colleagues [7] demonstrated identification under naturalistic viewing with portable EEG. Longitudinal work has further shown that EEG-based identity information can remain stable across repeated recordings separated by days to months [8,9], and deep-learning approaches have pursued session-invariant biometric representations [9,10]. In parallel, functional MRI research has shown that distributed brain connectivity patterns can identify individuals with high reliability, and subsequent work has framed identifiability itself as a measurable property of neuroimaging representations more broadly [11,16]. What remains less clear is how this biometric separability should inform the interpretation of routine EEG machine-learning studies in which identity is not the target.

### 1.2. Evaluation Validity and Structured Variance in M/EEG

That question is increasingly important because concerns about evaluation validity in M/EEG have become more explicit. COBIDAS MEEG and related good practice guidance has emphasised transparent reporting of preprocessing, split logic, and confound structure in order to improve reproducibility and interpretability [3,4]. One recurrent source of inflated performance is subject leakage, i.e., when segments from the same individual appear in both training and test sets, models can exploit stable person-specific features and inherit the associated target label without learning the intended cognitive or clinical relationship. This problem has been documented in translational EEG, digital health, and biometric benchmarking studies, among others [12,13,14]. Benchmark efforts such as M^3^CV have likewise highlighted the importance of separating cross-subject, cross-session, and cross-task sources of variation when evaluating EEG systems [17]. However, the link between these methodological concerns and the measurable size of subject-linked variance in a given EEG representation has remained mostly implicit. The present study seeks to make that link explicit by quantifying subject-linked structure directly and showing how it relates to both biometric discriminability and leakage-prone downstream evaluation.

## 2. Materials and Methods

### 2.1. Datasets

We analysed four publicly available EEG datasets selected to span a broad range of study designs, subject counts, recording systems, and experimental contexts. Together, they cover clinical resting-state EEG, low-channel consumer EEG, longitudinal repeated-session EEG, and large-scale research-grade task EEG. All datasets were reprocessed through a common feature-extraction pipeline so that subject-linked structure could be compared within a consistent representation. Table 1 summarises the key properties of the datasets and the role each one plays in the study.

*Mumtaz MDD.* The Mumtaz MDD dataset comprises resting-state clinical EEG from 63 subjects, including 33 patients with major depressive disorder and 30 healthy controls, recorded with a 19-channel system [18,19]. This dataset was used specifically for the leakage analysis because it provides a fixed subject-level diagnostic label, which makes it well suited for testing how segment-level splitting can inflate apparent clinical classification performance when subject identity is allowed to cross the train–test boundary. The same recordings have recently been used to demonstrate depression screening under a strictly leakage-free subject-wise protocol [20], providing an independent subject-level reference point for this dataset. To prevent identifier collisions between diagnostic groups, a unique subject key was constructed by combining the diagnostic label with the original numeric subject identifier.

*SignEEG v1.0.* The SignEEG dataset was acquired with a 5-channel Emotiv Insight consumer headset during genuine and forged signature imagery tasks, and originally reported recordings from 70 subjects [21]. It was included as a low-channel consumer-EEG case for examining whether subject-linked spectral structure transfers across a large paradigm shift under limited spatial sampling. However, the long numeric subject identifiers in the released feature table suffered floating-point precision loss during CSV export, which caused identifier collisions and reduced the number of uniquely resolved subjects from 70 to 39. All SignEEG analyses reported here therefore use 39 resolved subjects. Because unresolved collisions cannot be ruled out without re-processing the raw EDF files, the SignEEG results should be regarded as provisional and are used as a suggestive boundary-condition analysis rather than definitive evidence.

*Longitudinal ERP.* The longitudinal ERP dataset provides the main repeated-measures test bed for quantifying subject-linked variance and temporal persistence [22]. It contains 57-channel EEG recorded at 1000 Hz during an RSVP face-target task. A core repeated-measures subset, denoted Group A, includes 15 subjects recorded across four sessions on days 1, 7, 80, and 200, making it possible to estimate the relative contributions of subject and session-related variation over a 199-day span. The full dataset also contains an additional 380 single-session participants in Group B, yielding 395 uniquely identified subjects in total. In the present study, Group A was used for variance decomposition and cross-session analysis, while the full 395-subject dataset was used for biometric identification and verification experiments.

*PhysioNet EEGMMIDB.* The PhysioNet EEG Motor Movement/Imagery Database provides 64-channel research-grade EEG recorded at 160 Hz from 103 volunteers across 14 recording runs [23,24]. These runs include resting baselines and motor imagery conditions, which makes the dataset useful both for replicating the variance decomposition in an independent setting and for testing cross-paradigm transfer of subject identity across rest and task. Compared with the longitudinal ERP dataset, PhysioNet contributes a larger subject pool, more channels, and multiple repeated within-subject recording contexts.

All datasets were segmented into non-overlapping 2-s epochs after loading and harmonised through the same downstream preprocessing and feature-extraction steps described below. The resulting segment counts and retained channel counts varied by dataset, reflecting differences in study duration, sampling density, and montage size, but all analyses were conducted in a shared spectral feature space.

**Table 1:** Dataset properties. Segments are non-overlapping 2-s epochs. Ch denotes EEG channels retained. The SignEEG row lists 70 subjects as recorded; only 39 were uniquely resolved due to ID precision loss (see Section 2.1).

Dataset	Subj	Segments	Ch	Paradigm	Role
Mumtaz MDD [18,19]	63	8267	19	Resting-state, clinical	Leakage case
SignEEG v1.0 [21]	70 (39)	88,770	5	Signature imagery	Boundary condition
Longitudinal ERP [22]	395	71,307	57	RSVP face-target	Cross-session
PhysioNet [23,24]	103	81,870	64	Motor imagery	Large-scale

### 2.2. Preprocessing and Feature Extraction

All datasets were processed through a common pipeline designed to preserve comparability across studies while focusing on interpretable spectral features commonly used in EEG machine learning. Raw EEG signals were segmented into non-overlapping 2-s epochs. For each epoch and each channel, spectral density was estimated using Welch’s method with a window length of 256 samples or the segment length when shorter.

From these spectra, three families of features were derived. First, we computed log absolute band power in five canonical frequency bands: delta, 0.5–4 Hz; theta, 4–8 Hz; alpha, 8–13 Hz; beta, 13–30 Hz; and gamma, 30–45 Hz. Second, we computed relative band power by expressing band-limited power as a proportion of total power in the 0.5–45 Hz range within the same epoch. Third, we computed hemispheric asymmetry features as log right-minus-left band power for all available homologous electrode pairs. For the 5-channel SignEEG montage, the available homologous pairs were AF3–AF4 and T7–T8. For the remaining datasets, all standard 10–20 homologous pairs available in the retained montage were used. Depending on channel count and available asymmetry pairs, this yielded between 60 and 800 features per epoch.

Band-power values were obtained from linear-scale power spectral density estimates and log-transformed after band integration. Relative power was normalised within each epoch only and was not standardised across subjects. No cross-subject normalisation was applied at the feature construction stage. In experiments involving model fitting, any scaling was performed strictly within the training split, i.e., mean and variance were estimated from training data only and then applied to held-out data without re-fitting. This procedure prevented information from the evaluation set from influencing feature scaling.

No epoch rejection beyond the segmentation boundary was applied. All non-overlapping 2-s windows were retained regardless of amplitude. This choice prioritised a common and reproducible pipeline across datasets with heterogeneous acquisition properties. It also means that the subject-linked structure quantified here should be understood as a property of the measured spectral feature representation, potentially incorporating both neurophysiological and recording-related sources of individual specificity. The selected feature set follows prior EEG fingerprinting work that has emphasized interpretable spectral descriptors as a robust basis for subject discrimination [6].

### 2.3. Variance Decomposition

To quantify subject-linked structure directly, we performed a two-way variance components analysis on the repeated-measures portion of the longitudinal ERP dataset, using Group A only (15 subjects, 4 sessions, 630 spectral features). For each feature, observations were modelled as,

(1) $$x_{ijk}=\mu +\alpha_{i}+\gamma_{j}+\varepsilon_{ijk},$$

where μ is the grand mean, $\alpha_{i}$ is the subject-specific effect, $\gamma_{j}$ is the session effect, and $\varepsilon_{ijk}$ is residual variation at the epoch level. This formulation partitions feature variation into a between-subject component, a between-session component, and a residual component.

Variance components ${\hat{\sigma }}_{\alpha }^{2}$, ${\hat{\sigma }}_{\gamma }^{2}$, and ${\hat{\sigma }}_{\varepsilon }^{2}$ were estimated using the method of moments on subject-by-session cell means. The intraclass correlation coefficient ICC(2,1) was then computed for each feature as,

(2) $$\mathrm{ICC}\left(2,1\right)=\frac{{\hat{\sigma }}_{\alpha }^{2}}{{\hat{\sigma }}_{\alpha }^{2}+{\hat{\sigma }}_{\gamma }^{2}+{\hat{\sigma }}_{\varepsilon }^{2}},$$

and averaged across features to obtain a dataset-level summary of the proportion of spectral feature variance attributable to subject identity. Confidence intervals were estimated with 2000 bootstrap samples by resampling subjects with replacement and recomputing the mean ICC across features for each sample. This subject-level bootstrap propagates uncertainty at the unit of inference relevant to identity effects. ICC thresholds followed Koo and Mae [25], with values above 0.75 interpreted as excellent, 0.60–0.75 as good, and 0.40–0.60 as moderate.

The same analysis was replicated in the PhysioNet EEGMMIDB dataset, with recording run used as the repeated factor in place of session (103 subjects, 14 runs, 770 features). In both datasets, all subject-by-session or subject-by-run cells were populated, i.e., 407–548 epochs per cell in the longitudinal ERP data and 30–62 epochs per cell in PhysioNet. Although epoch counts varied modestly across cells, the estimator operated on cell means rather than raw epoch counts, so this imbalance was small relative to the variance components of interest. As a sensitivity analysis, the same variance decomposition was also estimated using a restricted maximum likelihood mixed-effects model with random intercepts for subjects and sessions or runs. The resulting ICC(2,1) estimate agreed with the method-of-moments value to two decimal places, supporting the robustness of the reported decomposition.

### 2.4. Biometric Evaluation Protocols

All biometric analyses were designed to isolate subject identity while preventing train–test leakage. Because extremely high verification performance can invite justified concern about evaluation artefacts, the split logic was made explicit and enforced at the subject level throughout. The verification pipeline is formalised as Algorithm 1. The core guarantees were fivefold. First, subjects were assigned to training or test groups before any downstream feature operation involving prototype construction or pair generation. Second, prototypes were formed separately within the training and test subject pools. Third, positive and negative pairs were generated independently within each pool, so that no pair ever mixed subjects from opposite sides of the split. Fourth, any feature normalisation was fit on training data only. Fifth, all verification metrics were computed only on subjects not used for model fitting.

Two biometric settings were evaluated, following the identification and verification distinction that is standard practice in conventional biometric modalities such as face recognition [26]. In closed-set identification, segments from each subject were split 70/30 into training and test partitions within subject. An Extra Trees classifier with 300 trees was trained on the training segments and evaluated on held-out segments across ten random repeats. This setting measures within-dataset subject discriminability and is intentionally easier than held-out-subject generalisation because all subjects appear in both training and test.

In verification, the protocol was stricter. Subjects were first divided into disjoint training and test groups using a 70/30 split at the subject level. Within each group, segments for each subject were divided into two halves, denoted A and B, and averaged to form subject prototypes. Same-subject positive pairs and different-subject negative pairs were then constructed independently within the training and test groups, using two negative pairs per positive pair. A learned verifier, implemented as an Extra Trees classifier operating on the absolute difference between paired feature vectors, was trained using training-group pairs only and evaluated using test-group pairs only. As a parameter-free reference, Euclidean distance between prototype vectors was also evaluated as a verification score baseline. Performance was summarised with area under the ROC curve and equal error rate across ten repeats.
**Algorithm 1** Held-out subject-split verification protocol**Input:** Dataset D with segments *x_ij_* for subjects *i* = 1,…, *N* and conditions *j*; split fractions *f*_tr_ = 0.7, *f*_te_ = 0.3; negative ratio *r* = 2
1:Partition subjects randomly into disjoint sets $S_{\mathrm{tr}}$ and $S_{\mathrm{te}}$ with $|S_{\mathrm{tr}}|/N\approx f_{\mathrm{tr}}$ ▹ Subject split is performed first, before any feature operation2:**for** g∈{tr,te} **do**3:   **for** each subject $i\in S_{g}$ **do**4:      Divide *i*’s segments into two equal halves $A_{i}$, $B_{i}$5:      Compute prototypes $p_{i}^{A}=\mathrm{mean}\left(A_{i}\right)$, $p_{i}^{B}=\mathrm{mean}\left(B_{i}\right)$6:   **end for**7:   $P_{g}^{+}\leftarrow \left\{\right|p_{i}^{A}-p_{i}^{B}\left|:i\in S_{g}\right\}$      ▹ Positive pairs: same subject, A vs. B prototype8:   Sample $r\;|P_{g}^{+}|$ pairs $\left(i_{1},i_{2}\right)$ with $i_{1}\neq i_{2}$, $i_{1},i_{2}\in S_{g}$9:   $P_{g}^{-}\leftarrow \left\{\right|p_{i_{1}}^{A}-p_{i_{2}}^{B}\left|\right\}$ ▹ Negative pairs formed independently within the same group10:**end for**11:Fit normalisation statistics and verifier V on $P_{\mathrm{tr}}^{+}\cup P_{\mathrm{tr}}^{-}$ only12:Evaluate AUC and EER of V on $P_{\mathrm{te}}^{+}\cup P_{\mathrm{te}}^{-}$ ▹ No subject in $S_{\mathrm{te}}$ was used at any earlier step
*Leakage-prevention guarantees: (i) subjects are split before any feature operation; (ii) prototypes are formed within each group only; (iii) no pair mixes subjects across the split; (iv) normalisation is fit on training data only; (v) metrics are computed on subjects unseen during training.*


### 2.5. Distance Decomposition, Cross-Session, Cross-Paradigm, and Leakage Experiments

Several additional analyses were used to characterise how subject-linked structure appears geometrically, how it persists over time, how it transfers across paradigms, and how it affects downstream evaluation.

For the distance decomposition, features were first standardised within the relevant analysis split. We then computed mean feature vectors for each subject under each available condition and calculated all pairwise Euclidean distances. These distances were partitioned into two classes, i.e., within-subject distances across sessions or conditions, and between-subject distances within the same session or condition. If subject identity forms a stable latent structure in the feature space, then within-subject cross-condition distances should be smaller than between-subject same-condition distances. This directional hypothesis was tested using a two-sided Mann–Whitney *U* test.

For cross-session identity analysis, we used the repeated-session Group A subset of the longitudinal ERP dataset. An Extra Trees classifier was trained on one session and tested on another, covering all six pairwise train–test session combinations. This analysis quantified how much subject-discriminative structure was retained as the time gap between training and test sessions increased.

For cross-paradigm analysis, classifiers were trained under one behavioural condition and evaluated under another while keeping subject identity as the target label. In PhysioNet, models were trained on resting baseline segments and tested on motor-task segments. In SignEEG, models were trained on genuine-signature imagery and tested on forged-signature imagery. These analyses examined whether subject-linked spectral structure was sufficiently robust to transfer across changes in paradigm.

For the leakage simulation, we used the Mumtaz MDD dataset to compare major depressive disorder classification under two evaluation schemes using a naive segment-level split and a correct subject-wise split. In both cases, classification was performed with logistic regression across ten repeats. Performance was computed both at the segment level and after averaging predicted probabilities within each test subject. To test whether subject identity alone was sufficient to account for the inflation under the naive protocol, we additionally trained a subject-identity classifier without clinical labels and mapped its subject-level predictions to diagnostic labels post hoc. This procedure provided a direct check on whether the observed inflation could be explained by identity leakage rather than by true biomarker learning.

## 3. Results

### 3.1. Variance Decomposition Shows That Identity Is the Largest Source of Structured EEG Variance

**Figure 1:**
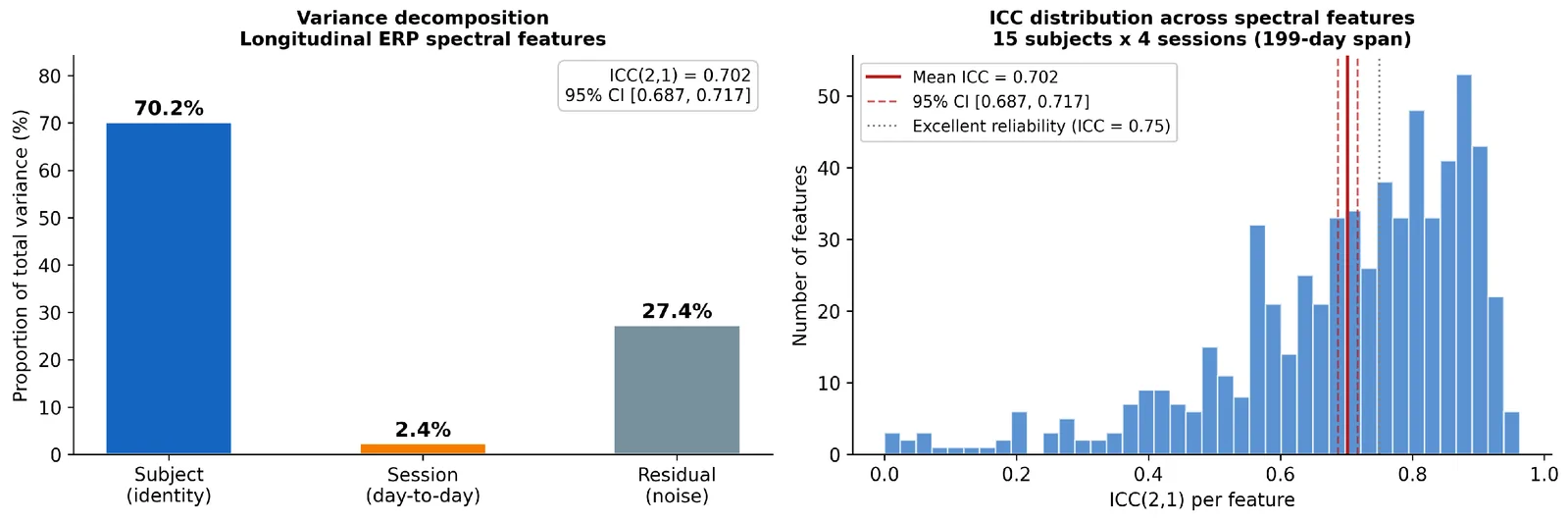
Variance decomposition for the longitudinal ERP dataset (Group A: 15 subjects, four sessions over 199 days, 630 spectral features). (**Left**) mean proportion of total variance attributable to subject identity (70.2%), session drift (2.4%), and residual noise (27.4%); annotation box shows ICC(2,1) with 95% bootstrap CI. (**Right**) distribution of ICC(2,1) across all 630 features; more than half exceed the excellent reliability threshold of ICC =0.75 (dotted line). Solid red line marks the mean ICC; dashed red lines indicate the 95% CI.

The variance decomposition showed that subject-linked structure dominated the spectral feature space in the repeated-session longitudinal dataset. Figure 1 and Table 2 summarise the results for the 15-subject Group A subset recorded across four sessions over 199 days. Subject identity accounted for 70.2% of total spectral feature variance, whereas session drift accounted for only 2.4% and the remaining 27.4% was attributed to residual variation. In practical terms, the between-subject component was approximately 29 times larger than the session-related component in this representation. The mean ICC(2,1) across the 630 features was 0.702, with a 95% bootstrap confidence interval from 0.609 to 0.737, placing the average feature in the good-to-excellent reliability range [25]. The median ICC was 0.748, indicating that more than half of all individual spectral features reached the conventional excellent-reliability threshold. The distribution of feature-wise ICC values was strongly right-skewed, with substantial mass at high values, showing that the subject effect was not driven by only a small subset of features but was expressed broadly across bands and channels. Because all 60 subject-by-session cells were fully populated, with 407 to 548 epochs per cell, these estimates were not driven by missing-cell structure.

This pattern replicated in an independent dataset with a different population, task structure, and acquisition protocol. Table 3 shows the same variance decomposition in the PhysioNet EEGMMIDB dataset, where 103 subjects contributed 14 runs each and 770 spectral features were analysed. Here, subject identity accounted for 80.7% of total variance, compared with 2.9% for run-to-run drift, yielding a mean ICC(2,1) of 0.807 with a 95% bootstrap confidence interval from 0.778 to 0.822. The median ICC was 0.836. Across the longitudinal ERP and PhysioNet datasets, subject identity therefore explained roughly 70–81% of the variance in the spectral representation and exceeded session- or run-level effects by a factor of about 24 to 28. The consistency of this pattern across different channel counts, paradigms, and repeated-measures designs supports the conclusion that subject-linked variance is a dominant component of common EEG spectral features rather than an isolated property of a single dataset.

### 3.2. Within-Subject Versus Between-Subject Geometry

The variance decomposition implies that the spectral feature space should be organised so that repeated measurements from the same individual remain closer to one another than measurements from different individuals. The distance analysis supported that expectation in three of the four datasets (Table 4). In the Mumtaz MDD, longitudinal ERP, and PhysioNet datasets, within-subject cross-session or cross-condition distances were all substantially smaller than between-subject distances measured within the same session or condition, and each contrast was highly significant under the two-sided Mann–Whitney *U* test. The effect was especially pronounced in the longitudinal ERP and PhysioNet datasets, where the within-to-between distance ratio was 0.426 in both cases despite substantial differences in population, montage, and task. Whether that numerical agreement reflects a deeper regularity of the spectral representation or is merely coincidental cannot be determined from the present data, but in both datasets it indicates that repeated within-subject structure occupies a much tighter region of feature space than between-subject variation.

**Figure 2:**
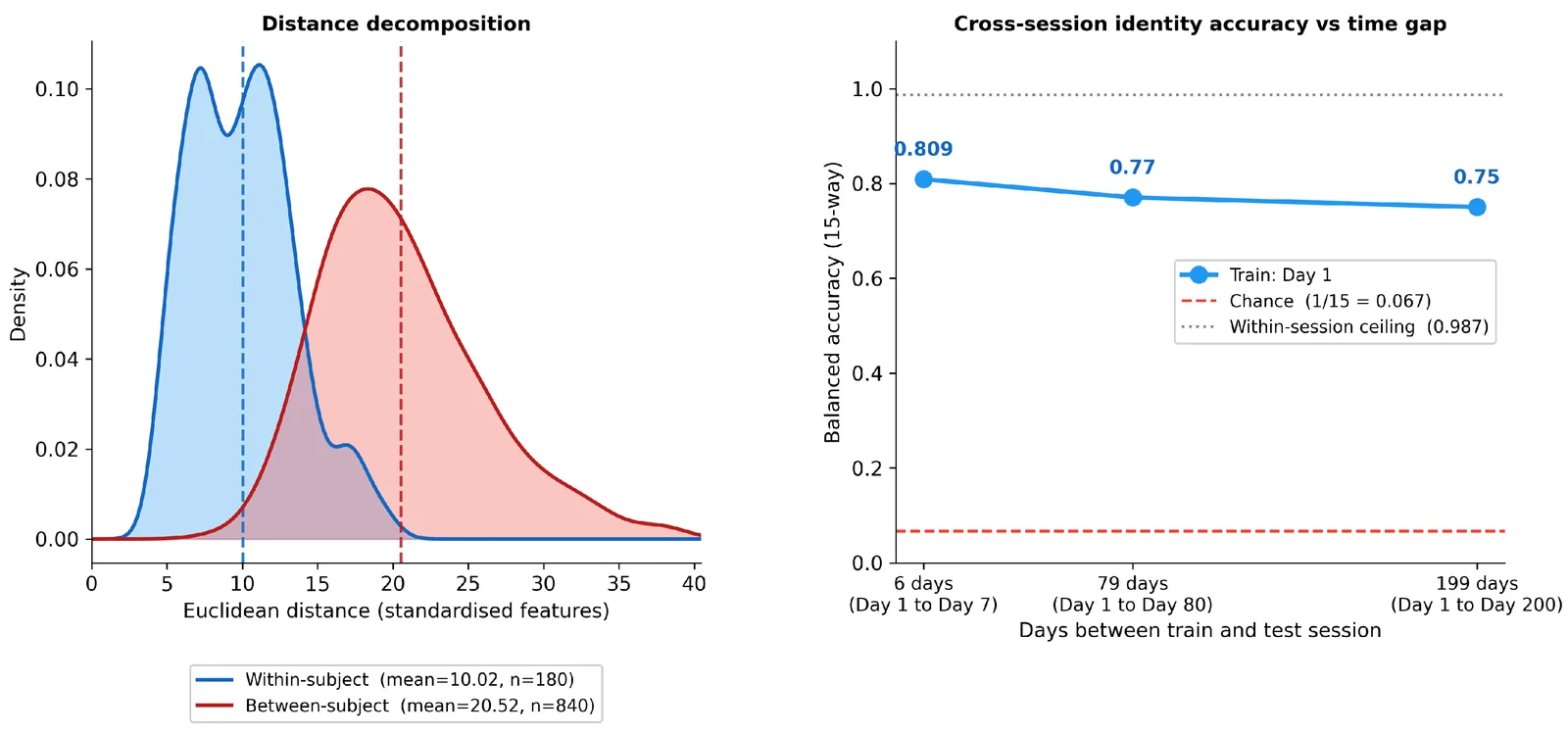
Subject identity as a stable latent factor in EEG. (**Left**) KDE distributions of within-subject cross-session distances (blue, mean =10.02, n=180) and between-subject same-session distances (red, mean =20.52, n=840) for the longitudinal ERP dataset. Within-subject distances are less than half of between-subject distances (ratio 0.43, p<0.0001, two-sided Mann–Whitney *U*). (**Right**) balanced accuracy for Day 1-trained cross-session classifiers plotted against the time gap. The dotted line shows the within-session ceiling (0.987); the dashed red line shows chance (0.067). Accuracy declines by only 5.9 percentage points over 199 days.

The longitudinal ERP dataset illustrates this geometry most clearly. Figure 2 (left panel) shows the full distance distributions. The mean within-subject cross-session distance was 10.02, whereas the mean between-subject same-session distance was 20.52. Thus, measurements from the same individual across sessions were, on average, less than half as far apart as measurements from different individuals within the same session. This pattern is consistent with the large subject-linked variance component observed in the decomposition analysis.

SignEEG was the only exception. In that dataset, the mean within-subject cross-paradigm distance slightly exceeded the mean between-subject distance, producing a ratio of 1.027 and a non-significant test result. This inversion is consistent with the cross-paradigm transfer failure reported below, suggesting that in the low-channel consumer-EEG setting the shift from genuine to forged signature imagery may alter the spectral representation enough to overwhelm person-specific structure. However, because the SignEEG subject identifiers were affected by precision-loss collisions, that result should be regarded as suggestive rather than definitive.

### 3.3. Biometric Identification and Verification

The same subject-linked structure that appeared in the variance and distance analyses also supported strong biometric discriminability across all four datasets. Table 5 shows closed-set identification performance under within-subject segment splits. Balanced accuracy was 0.939 in Mumtaz MDD, 0.699 in SignEEG, 0.405 in the much harder 395-way longitudinal ERP problem, and 0.941 in PhysioNet. When converted to multiples of chance, these correspond to approximately 59-fold, 27-fold, 162-fold, and 97-fold performance above chance, respectively. The longitudinal ERP dataset is particularly informative here. Although the balanced accuracy was lower in absolute terms because of the very large number of candidate identities, top-5 accuracy reached 0.913, indicating that the correct subject was among the top five predicted candidates in the large majority of trials. These identification results confirm that the spectral feature space is highly person-discriminative within each dataset.

These identification experiments should be interpreted as descriptive evidence of separability rather than as a test of held-out-subject generalisation. Because segments from every subject were split within subject into training and test subsets, the same identities were present on both sides of the evaluation. The task is, therefore, intentionally easier than downstream EEG prediction problems in which identity is a confound rather than the target. That distinction matters, i.e., the high identification performance is valuable here precisely because it reveals how much stable subject-linked information is available for a model to exploit if evaluation is not controlled appropriately.

Verification under the stricter held-out subject protocol was even more consistent. Table 6 shows that the learned verifier achieved an AUC of 1.000 with zero observed variance across repeats in the Mumtaz MDD, SignEEG, and PhysioNet datasets, and 0.999 in the 395-subject longitudinal ERP dataset. Even the parameter-free Euclidean distance baseline achieved AUC values of at least 0.982 in every dataset. These results indicate that subject identity in the spectral feature space was so strong that it could be recovered with near-perfect reliability even when training and test subjects were completely disjoint. Because the verification pipeline prevented any mixing of subjects or scaling statistics across the split boundary, the observed performance reflects genuine identity separability rather than artefactual leakage in the verification procedure itself.

### 3.4. Temporal Persistence and Paradigm-Invariance Boundary Conditions

The repeated-session longitudinal dataset allowed the stability of subject-linked structure to be tested explicitly over time. Figure 2 (right panel) and Table 7 show cross-session identity classification across all six session-pair combinations in Group A. Performance remained well above the chance level of 0.067 in every case, even when training and test sessions were separated by many months. The highest cross-session accuracy was 0.820 for the Day 7 to Day 80 comparison, and the most temporally demanding contrast, training on Day 1 and testing on Day 200, still achieved a balanced accuracy of 0.750. Relative to the within-session Day 1 ceiling of 0.987, this corresponds to a retention of 76.0% after 199 days. Across the full set of comparisons, retention ranged from 76.0% to 83.1%. Notably, the decline from the 6-day gap to the 199-day gap was only 5.9 percentage points. This degree of persistence is consistent with the variance decomposition result, i.e., when subject-linked variation dominates session drift in the spectral representation, strong identity recovery across time is expected.

We next asked whether the same subject-linked structure transfers across paradigms rather than across time alone. Table 8 compares cross-condition identity transfer in PhysioNet and SignEEG. In the 64-channel PhysioNet data, identity classifiers trained on resting baseline segments and tested on motor-task segments achieved a balanced accuracy of 0.504, corresponding to 51.9 times chance. Thus, substantial subject-discriminative information survived the shift from rest to motor imagery in the higher-density research-grade setting. In contrast, the SignEEG classifier trained on genuine-signature imagery and tested on forged-signature imagery achieved a balanced accuracy of only 0.016, or 0.6 times chance, which is below chance level. This contrast suggests a boundary condition, i.e., identity-linked spectral structure is not necessarily paradigm-invariant, and its transfer may depend on both recording richness and the cognitive distance between conditions. The rest-to-motor shift in PhysioNet represents a moderate state change in a 64-channel montage, whereas the genuine-to-forged signature contrast in SignEEG likely involves a much larger shift in attentional, visuomotor, and planning demands under a far sparser 5-channel montage. This interpretation is also consistent with the distance analysis, where SignEEG was the only dataset in which within-subject cross-condition distances were not smaller than between-subject distances. Because of the identifier issue in SignEEG, this low-channel boundary-condition result should be treated as provisional.

### 3.5. Leakage Simulation: Identity as the Mechanism

The most direct consequence of strong subject-linked structure is that it can inflate downstream prediction when evaluation is performed at the segment level rather than the subject level. Table 9 demonstrates this effect in the Mumtaz MDD major depressive disorder dataset. Under the naive segment-level split, logistic regression achieved an AUC of 0.9996 at the segment level and 1.000 at the subject level. Under the correct subject-wise split, performance dropped to 0.939 at the segment level and 0.959 at the subject level. The naive protocol therefore inflated AUC by 0.061 at the segment level and by 0.041 at the subject level, with both differences statistically significant.

The identity-derived control analysis clarifies the mechanism of this inflation. A classifier trained only to recognise subjects from spectral features, without access to clinical labels during training, achieved a subject-level MDD AUC of 1.000 once its subject-identity predictions were mapped to diagnosis labels post hoc. In other words, subject identity alone was sufficient to recover the clinical grouping perfectly in this dataset. This shows that the inflated performance under the naive split can be explained by identity leakage without invoking superior biomarker learning. The result does not imply that no genuine MDD-related signal is present; indeed, the correct subject-wise split still yielded high performance, consistent with recent subject-wise depression-screening results on the same recordings [20]. Rather, it shows that naive evaluation obscures the boundary between a clinically meaningful classification problem and a much easier subject-recognition problem when diagnosis is fixed within person.

A second feature of the leakage analysis is the difference in uncertainty between protocols. Performance under the naive split was not only higher but also appeared much more stable, with a segment-level standard deviation of 0.0004 across repeats. Under the subject-wise split, the standard deviation increased to 0.045. The narrow dispersion in the naive setting is, therefore, misleading. It reflects the ease and determinism of the confounded problem, not superior confidence in clinically relevant generalisation. The permutation result further supports this interpretation, with shuffled subject-level labels yielding a significant test outcome. Taken together, these analyses identify subject-linked spectral structure as a sufficient and likely dominant mechanism of performance inflation in this leakage-prone clinical setting.

## 4. Discussion

The main contribution of this study is methodological. In the spectral EEG feature representations examined here, subject identity is a major source of structured variance and a plausible source of performance inflation when training and test data are not separated at the subject level. The key implication is not simply that EEG supports biometric discrimination, but that the same subject-linked structure can confound decoding and clinical classification if the evaluation is naive.

This point matters because good predictive performance does not, by itself, show that a model has learned the intended cognitive or clinical signal. In spectral band-power representations, models may also exploit stable person-specific structure unrelated to the target variable. The relevant methodological question is therefore whether the evaluation design prevents subject information from crossing the train/test boundary [3,4].

Our results also suggest that subject identity should be interpreted as a property of the *representation* studied here rather than as a universal property of all EEG analyses. The dominance of subject-linked variance was shown for spectral band-power and asymmetry features, and it may differ for other representations. EEG machine learning encompasses a broad range of feature families and classifier architectures [27], and confound structure should be expected to depend in part on the chosen analysis pipeline. Within the spectral band-power family studied here, however, the effect was substantial, replicated in an independent dataset, and persistent across sessions over several months.

The cross-paradigm and leakage analyses clarify the practical limits of this structure. Identity transferred strongly in high-channel research-grade EEG, but not in the provisional low-channel SignEEG analysis, suggesting that its expression depends on recording richness and task contrast. More importantly, in the MDD example, naive segment-level evaluation inflated apparent classification performance, and subject identity alone was sufficient to recover diagnostic labels. This shows how a clinical classification problem can collapse into a subject-recognition problem when labels are fixed within a person.

The practical implication is straightforward. For between-subject EEG decoding and clinical classification, subject-wise train/test separation should be the default. Studies using spectral EEG features should also report diagnostics of subject-linked variance and describe evaluation protocols with enough precision for leakage risk to be assessed.

Several limitations qualify these findings. The main variance decomposition was based on 15 repeatedly measured subjects in one longitudinal paradigm, although the central pattern replicated in PhysioNet. The analyses were restricted to spectral band-power and asymmetry features, so other feature families may show different variance partitions. Covariance-based and Riemannian approaches, for instance, operate in a substantially different representational geometry and may exhibit different subject-to-session variance ratios [28]. Because all epochs were retained after segmentation, the measured subject-linked structure should not be interpreted as purely neural; it may include recording-related as well as neurophysiological components, a concern that extends to EEG machine-learning more broadly [29]. Finally, the SignEEG boundary-condition result remains provisional because of subject-identifier corruption in the released feature table.

## 5. Conclusions

This study shows that subject identity is a dominant source of structured variance in spectral EEG feature representations, and that it has direct consequences for evaluation validity in EEG machine learning. In a longitudinal dataset spanning 199 days, subject-related variance greatly exceeded session-related drift, and this result replicated in an independent large-scale dataset. Across four datasets, the same subject-linked structure supported very strong biometric separability and substantial cross-session persistence, indicating that common spectral features contain stable information that distinguishes individuals over time. At the same time, the expression of this structure was not fully invariant across recording contexts, appearing stronger in richer high-channel recordings than in low-channel consumer EEG under larger paradigm shifts.

The methodological implication is clear. When subject-linked structure is strong in the feature space, evaluation protocols that do not separate training and test data at the subject level can produce inflated estimates of downstream performance. Our leakage analysis illustrates this directly. Apparent clinical classification performance increased under naive segment-level splitting, and subject identity alone was sufficient to recover diagnostic labels in that setting. The issue is therefore not only that EEG contains individual fingerprints, but that these fingerprints can be unintentionally exploited by predictive models when evaluation is not designed to control them.

Thus, these findings support treating subject identity as a measurable confound risk in spectral EEG studies rather than as a background nuisance. For studies using comparable feature representations, subject-wise evaluation should be the default, and explicit diagnostics of subject-linked variance should accompany downstream decoding or clinical classification results. More broadly, quantifying structured between-subject variance offers a practical route toward more interpretable, reproducible, and methodologically robust M/EEG machine learning research. 

## Figures and Tables

**Table 2:** Variance components and ICC(2,1) for the longitudinal ERP dataset (Group A, 630 features). Range shows the 5th–95th percentile across features.

Component	Mean Proportion (%)	Range (5th–95th Pct, %)
Subject identity $\left({\hat{\sigma }}_{\alpha }^{2}\right)$	**70.2**	32.7–91.3
Session drift $\left({\hat{\sigma }}_{\gamma }^{2}\right)$	2.4	0.0–8.2
Residual noise $\left({\hat{\sigma }}_{\varepsilon }^{2}\right)$	27.4	7.9–62.2
ICC(2,1) mean	0.702 (95% CI: 0.609–0.737)	
ICC(2,1) median	0.748	

**Table 3:** Variance decomposition replication in the PhysioNet EEGMMIDB dataset (103 subjects, 14 runs, 770 spectral features). Run-to-run drift is the repeated factor (analogous to session in the longitudinal dataset). All 1442 subject-run cells are fully populated (30–62 epochs each). The bootstrap CI resamples subjects with replacement.

Component	Mean Proportion (%)	Range (5th–95th Pct, %)
Subject identity $\left({\hat{\sigma }}_{\alpha }^{2}\right)$	**80.7**	–
Run-to-run drift $\left({\hat{\sigma }}_{\gamma }^{2}\right)$	2.9	–
Residual $\left({\hat{\sigma }}_{\varepsilon }^{2}\right)$	16.3	–
ICC(2,1) mean	0.807 (95% CI: 0.778–0.822)	
ICC(2,1) median	0.836	

**Table 4:** Distance decomposition (mean ± SD of Euclidean distances in the standardised feature space). Within: same subject across sessions or conditions. Between: different subjects in the same session or condition. Ratio <1.0 confirms within-subject distances are smaller. *p*: two-sided Mann-Whitney *U* test.

Dataset	Within-Subject	Between-Subject	Ratio	*p*
Mumtaz MDD	14.03±4.14	20.41±6.27	0.688	<0.0001
SignEEG	5.38±1.51	5.24±1.54	1.027	0.840
Longitudinal ERP	8.74±3.01	20.49±8.63	0.426	<0.0001
PhysioNet	11.11±7.41	26.08±9.57	0.426	<0.0001

**Table 5:** Closed-set identification (ten repeats, mean ± SD). Segments are split 70/30 within each subject; all subjects appear in both training and test. Ratio is balanced accuracy divided by chance.

Dataset	*N*	Balanced Acc	Top-5 Acc	Chance	Ratio
Mumtaz MDD	63	0.939±0.002	0.953±0.003	0.016	59×
SignEEG	39	0.699±0.003	0.912±0.002	0.026	27×
Longitudinal ERP	395	0.405±0.003	0.913±0.001	0.003	162×
PhysioNet	103	0.941±0.002	0.988±0.001	0.010	97×

**Table 6:** Verification performance (ten repeats, mean ± SD) under the correct subject-split held-out protocol. Distance: Euclidean distance-based. Learned: Extra Trees on the absolute feature difference. EER: equal error rate.

Dataset	Distance-Based		Learned Verifier	
	AUC	EER	AUC	EER
Mumtaz MDD	0.982±0.011	0.070	1.000±0.000	0.000
SignEEG	1.000±0.000	0.000	1.000±0.000	0.000
Longitudinal ERP	0.995±0.004	0.006	0.999±0.002	0.006
PhysioNet	1.000±0.000	0.000	1.000±0.000	0.000

**Table 7:** Cross-session identity classification (longitudinal ERP, Group A, 15 subjects). Retention is cross-session balanced accuracy as a proportion of the within-session ceiling of 0.987.

Train	Test	Gap (Days)	Balanced Acc	Retention
Day 1	Day 7	6	0.809	81.9%
Day 1	Day 80	79	0.770	78.0%
Day 1	Day 200	199	**0.750**	76.0%
Day 7	Day 80	73	0.820	83.1%
Day 7	Day 200	193	0.805	81.6%
Day 80	Day 200	120	0.780	79.0%
Within-session (Day 1)		—	0.987	100%

**Table 8:** Cross-paradigm identity transfer (ten repeats, mean ± SD). All subjects appear in both training and test; only recording condition is held out. Ratio is balanced accuracy divided by chance.

Dataset	Train Condition	Test Condition	*N*	Balanced Acc	Ratio
PhysioNet	Baseline (rest)	Motor task	103	0.504±0.001	51.9×
SignEEG	Genuine signing	Forged signing	39	0.016±0.001	0.6×

**Table 9:** MDD classification under two evaluation protocols (Mumtaz MDD dataset, logistic regression, ten repeats). Segment-level AUC is computed directly from per-segment predicted probabilities; subject-level AUC averages segment probabilities within each test subject before computing ROC, which is the appropriate evaluation unit. The identity-derived AUC maps predicted subject-identity probabilities to clinical labels without access to clinical labels during training. All *p*-values are two-sided.

Protocol	Level	AUC (Mean ± SD)	Inflation	*p* (Two-Sided)
Naive (segment split)	Segment	0.9996±0.0004	+0.061	0.0002
Naive (segment split)	Subject	1.000±0.000	+0.041	0.0022
Correct (subject split)	Segment	0.939±0.045	—	—
Correct (subject split)	Subject	0.959±0.031	—	—
*Identity-derived AUC* (subject level, no clinical labels): 1.000				
*Permutation test* (N=200, labels shuffled at subject level): p<0.005				

## Data Availability

All datasets analysed in this study are publicly available. The Mumtaz MDD dataset: https://doi.org/10.6084/m9.figshare.4244171 (accessed on 14 September 2026); SignEEG v1.0: https://zenodo.org/records/8332198 (accessed on 14 September 2026); Longitudinal ERP: https://springernature.figshare.com/articles/dataset/27201003 (accessed on 14 September 2026); PhysioNet EEGMMIDB: https://www.physionet.org/content/eegmmidb/1.0.0/ (accessed on 14 September 2026). The complete codebase, along with processed data to replicate all results reported here, is available at https://github.com/ugail/eeg-subject-identity-study (accessed on 14 September 2026).
